# Why mother rats protect their children

**DOI:** 10.7554/eLife.28514

**Published:** 2017-06-13

**Authors:** Ksenia Z Meyza, Ewelina Knapska

**Affiliations:** Laboratory of Emotions Neurobiology, Nencki Institute of Experimental Biology, Warsaw, Poland; Laboratory of Emotions Neurobiology, Nencki Institute of Experimental Biology, Warsaw, Polande.knapska@nencki.gov.pl

**Keywords:** maternal behavior, freezing, social interactions, learning, Rat

## Abstract

The presence of the hormone oxytocin in the central amygdala makes a mother rat willing to put her life in danger in order to protect her offspring.

**Related research article** Rickenbacher E, Perry RE, Sullivan RM, Moita MA. 2017. Freezing suppression by oxytocin in central amygdala allows alternate defensive behaviours and mother-pup interactions. *eLife* **6**:e24080. doi: 10.7554/eLife.24080

When an animal encounters a threat it has to analyze both the threat (what type of threat is it? how close is it?) and also the local environment (can I escape? is there anywhere to hide?). The animal must then choose from a range of possible responses: it can, for example, try to fight the threat or to escape (Blanchard et al., 2011). Another possibility, when there is no safe escape route, is to 'freeze' and hope that a predator will not notice you (Blanchard and Blanchard, 1969). Freezing can be a viable strategy when an animal is on its own, but it is not an option for a mother looking after young offspring (Figure 1). With very young progeny that cannot run, the only choice is to confront the threat. When the offspring are a bit older, it might be possible to usher them to safety.

**Figure 1.**
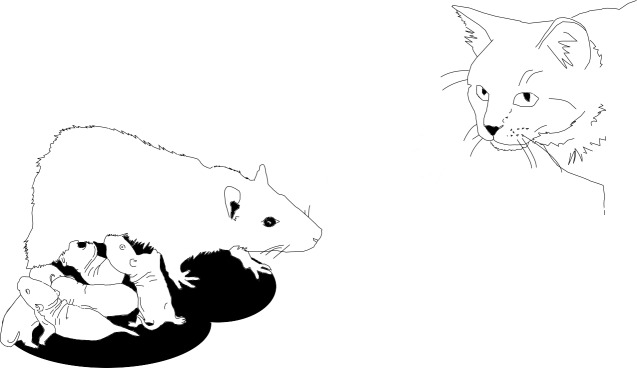
The way a mother rat responds to a threat depends on the age of her pups.

Illustration: Karolina Rokosz.

While the neural mechanisms that give rise to different defensive responses are relatively well understood, the vast majority of studies reported to date have been based solely on experiments on male subjects. Moreover, the parental status of the animals was often neglected. Female rats are generally considered less territorial than males, but their behavior changes once they become mothers (also known as 'dams') and they can become aggressive towards potentially dangerous intruders, even if this will also place the dam in danger (Bosch, 2013). How does the brain switch between self-defense and offspring-defense modes? Is the defensive repertoire affected by the age of the pups? Now, in eLife, Marta Moita and colleagues – including Elizabeth Rickenbacher as first author, Rosemarie Perry and Regina Sullivan – report that, in the presence of pups, self-defense responses are suppressed by the presence of a hormone called oxytocin in a region of the brain called the central amygdala (Rickenbacher et al., 2017).

Oxytocin is a well known hormone that promotes social bonding and causes contractions of the uterus and cervix during sexual intercourse and childbirth, as well as milk ejection during breastfeeding. More recently it was discovered that oxytocin can also control freezing behavior (Knobloch et al., 2012). In general oxytocin is released into the blood. However, in the case of the fear response, oxytocin is secreted directly into the central amygdala, which is one of the structures in the brain that controls freezing behavior (Wilensky et al., 2006).

In a series of elegant experiments Rickenbacher et al. show that, in the presence of pups, dams do not freeze when confronted with a threat (here, a noxious stimulus delivered along with an odor). Moreover, their response depends on the age of the pups. With very young pups (between four and six days old), the dams approach the threat. However, if the pups are older (between 19 and 21 days old), the dams turn towards the pups and huddle with them (probably because older pups are capable of running away if necessary).

This pattern of behavior changes dramatically if an oxytocin antagonist is very precisely injected into the central amygdala. When the oxytocin is blocked by the antagonist, the dams start behaving as if the pups were not present and they often freeze when exposed to threatening situations.

This change in the behavior of the dams has important implications for the pups. In a normal situation, a dam does not freeze in the presence of her pups and instead displays an array of active defensive behaviors. During this process, the pups learn to associate the noxious stimulus and odor, which are intended for the mother, with something unpleasant. If the mothers freeze, this emotional information is not transferred from the dam to the pups.

The work of Rickenbacher et al. – who are based at the Champalimaud Neuroscience Programme in Portugal and New York University School of Medicine – addresses some important questions, and also raises new ones. Is it just the oxytocin in the central amygdala that suppresses freezing of the dams? How do the pups learn about the danger? Answering these questions will keep neuroscientists (and rats) busy for years to come.
